# Geometry-Aware Enhanced 6DRepNet for Single-RGB Head Pose Estimation

**DOI:** 10.3390/s26103266

**Published:** 2026-05-21

**Authors:** Hua Yang, Yuanyuan Li, Mingzhi Mu, Ming Zhao

**Affiliations:** College of Mathematics and Computer Science, Wuhan Polytechnic University, Wuhan 430040, China; xiaomumu1203@gmail.com (M.M.); zhaoming202307@163.com (M.Z.)

**Keywords:** head pose estimation, 6DRepNet, 6D rotation representation, single-RGB, geometric consistency, geodesic loss

## Abstract

Head pose estimation is a fundamental task in facial analysis and behavior understanding. To address the limitations of 6DRepNet in single-RGB scenarios, particularly in high-level spatial discriminative region modeling, global feature-to-6D rotation representation mapping, and the optimization of large-pose challenging samples, this paper proposes a geometry-aware enhanced framework for head pose estimation. While preserving the 6D continuous rotation representation and its SO(3)-based geometric supervision mechanism, the proposed method improves the baseline model through the joint design of a Spatial Recalibration Module, a Residual Pose Mapping Head, and a Pose-Aware Weighted Geodesic Loss. Experiments are conducted using 300W-LP for training and AFLW2000 and BIWI for evaluation. The results show that the proposed method consistently outperforms the baseline 6DRepNet on both datasets, reducing the overall MAE from 3.97 to 3.72 on AFLW2000 and from 3.54 to 3.26 on BIWI. Ablation studies further verify the effectiveness and complementarity of the proposed components. These results demonstrate that the proposed method can effectively improve the accuracy and robustness of single-RGB head pose estimation without introducing additional modalities.

## 1. Introduction

Head pose estimation (HPE) aims to estimate the three-dimensional orientation of the human head from a single image or a video sequence, usually represented by yaw, pitch, and roll angles [1]. As an important foundation for facial analysis and behavior understanding, head pose estimation has wide applications in driver monitoring, human-computer interaction, classroom behavior analysis, intelligent surveillance, and virtual reality [2,3,4]. However, in unconstrained scenarios, factors such as illumination variation, partial occlusion, background interference, reduced image resolution, and large pose changes can significantly weaken feature discriminability, thereby increasing the difficulty of head pose estimation.

For single RGB-based HPE tasks, Hempel et al. proposed 6DRepNet [5], which employs a continuous 6D rotation representation optimized with a geodesic loss. This approach demonstrates advantages in pose representation continuity, rotational space constraints, and geometric consistency [6]. Nonetheless, 6DRepNet still has room for improvement. First, the original model directly maps global features to 6D pose using a single linear layer after global average pooling, which limits the modeling capacity for complex nonlinear relationships between high-level semantic features and pose parameters [7]. Second, the original model lacks an explicit mechanism to model spatially discriminative regions, making it difficult to highlight key facial areas under complex backgrounds, occlusions, or insufficient facial cues [8,9]. Third, the standard geodesic loss applies uniform supervision to all samples, without assigning stronger constraints to large-pose or hard samples.

In recent years, the development of Transformer-based and multimodal approaches has further improved the robustness and generalization of head pose estimation (HPE). For example, methods such as TokenHPE, TokenFOE, CogVLM, and SemiUHPE leverage attention mechanisms and multimodal feature embeddings to achieve robust estimation under challenging head poses [10,11,12,13]. In addition, advanced deep learning techniques in other vision tasks have demonstrated remarkable progress. For instance, TTST and EDiffSR employ Transformers and diffusion models to capture complex spatial and temporal relationships, highlighting the potential of high-level feature representations for visual tasks [14,15]. Although these recent Transformer-based and vision-language-model-based methods have achieved promising performance, a 6DRepNet-based framework remains a practical and meaningful choice for single-RGB head pose estimation. This is mainly because 6DRepNet provides a compact architecture, a continuous 6D rotation representation, and SO(3)-based geodesic supervision, which together ensure stable geometric consistency during pose regression. Compared with large-scale Transformer or multimodal models, 6DRepNet is easier to train and deploy in real-time or resource-constrained scenarios while maintaining competitive estimation accuracy. Therefore, this work focuses on improving the representational and optimization limitations of 6DRepNet rather than replacing it with a more complex model.

To address the limitations of 6DRepNet under single-RGB settings, this paper proposes a geometry-aware enhancement framework for HPE. Structurally, a Spatial Recalibration Module (SRM) is introduced to enhance the response of pose-sensitive regions while suppressing background interference, and a Residual Pose Mapping Head (RPMH) is designed to improve the nonlinear mapping from high-level semantic features to 6D rotation representations. Optimally, a Pose-Aware Weighted Geodesic Loss is proposed, which assigns higher training weights to large-pose and difficult samples. By combining SRM for spatial attention enhancement, RPMH for nonlinear feature-to-pose mapping, and the weighted geodesic loss for prioritizing challenging samples, the three components work synergistically to significantly improve overall head pose estimation performance. Experimental results on the AFLW2000 and BIWI datasets demonstrate that the proposed method outperforms the baseline 6DRepNet across all metrics, and ablation studies further confirm the effectiveness and complementarity of each module.

The main contributions of this work are summarized as follows:

1. A geometry-aware HPE framework is developed to address the limitations of 6DRepNet in spatially discriminative modeling, pose representation mapping, and difficult-sample optimization under single-RGB settings.
2. Lightweight structural enhancements, including SRM and RPMH, are proposed to improve spatial attention and 6D rotation regression capabilities without introducing additional modalities.
3. A Pose-Aware Weighted Geodesic Loss is designed to assign higher optimization weights to large-pose and difficult samples, enhancing the learning of complex pose instances.
4. Extensive experiments on the AFLW2000 and BIWI datasets demonstrate that the proposed method outperforms the baseline, and ablation studies validate the effectiveness and complementarity of the proposed modules.

## 2. Related Work

Recent head pose estimation methods can be broadly categorized into keypoint-based and keypoint-free approaches. Keypoint-based methods detect facial landmarks and combine them with 3D head models to recover poses, but they are prone to error accumulation under occlusions, low-resolution images, or large-angle rotations [16]. In contrast, keypoint-free methods learn the mapping from images to head poses directly, reducing dependency on landmark detection quality and becoming the mainstream in recent research. Among keypoint-free approaches, one line treats head pose estimation as a classification or regression problem over Euler angles, such as HopeNet [17], WHENet [18], and FSA-Net [19]. Another line introduces more geometrically constrained rotation representations, such as QuatNet [20] and TriNet [21], leveraging quaternions or rotation matrices to enhance geometric consistency. A third line focuses on improving feature representation under different pose conditions to boost discriminability, such as FDN [22]. While these methods outperform traditional keypoint-based approaches in complex scenes, they remain sensitive to discontinuities in Euler angles, face optimization constraints, or exhibit limited feature discriminability [23].

To alleviate these issues, 6DRepNet proposes a continuous 6D rotation representation combined with geodesic loss, improving rotational space consistency and mitigating the effects of Euler angle discontinuities and gimbal lock. However, the original 6DRepNet still employs a single linear layer after global average pooling, limiting its ability to model complex nonlinear relationships between high-level semantic features and pose parameters. It also lacks explicit spatial discrimination mechanisms and insufficiently emphasizes large-pose or difficult samples. The recent development of Transformer and multi-modal methods further enhances HPE robustness and generalization. Methods like TokenHPE, TokenFOE, CogVLM, and SemiUHPE exploit attention mechanisms and multi-modal embeddings for robust estimation under complex poses. Additionally, deep networks in other vision tasks, such as TTST and EDiffSR, capture complex spatiotemporal dependencies, demonstrating the potential of high-level feature representations.

Against this backdrop, this work proposes three complementary modules—SRM, RPMH, and Pose-Aware Weighted Geodesic Loss—on top of 6DRepNet, enhancing feature modeling, pose mapping capability, and training objectives, collectively boosting the head pose estimation performance in single-RGB scenarios.

## 3. Materials and Methods

### 3.1. Datasets and Data Preprocessing

#### 3.1.1. Datasets

The proposed model is trained on the 300W-LP dataset and evaluated on the AFLW2000 and BIWI datasets. The 300W-LP dataset is an extended version of the 300W dataset generated through the face profiling technique. It contains 61,225 face images with large pose variations and provides corresponding landmark annotations. Owing to its broad pose coverage, it has been widely used as a training dataset for monocular RGB head pose estimation, and is therefore adopted in this work for model training.

During the testing stage, the AFLW2000-3D dataset is first used for performance evaluation. This dataset contains 2000 images and provides 68 three-dimensional facial landmark annotations for each sample. Since it exhibits diverse pose variations and many images are collected in natural scenes with occlusion, illumination changes, and large-pose challenges, AFLW2000 is commonly used as a standard benchmark for evaluating head pose estimation methods under unconstrained conditions.

In addition, the BIWI dataset is used to further evaluate the effectiveness of the proposed method under controlled conditions. BIWI contains more than 15,000 RGB-D images with a resolution of 640 × 480, collected from 20 subjects, including 6 females and 14 males. The dataset covers a large pose range, with yaw angles of approximately ±75° and pitch angles of approximately ±60°, and provides RGB images, depth images, as well as ground-truth annotations of head position and rotation for each frame. Since this work focuses on single-RGB head pose estimation, only the RGB images and the corresponding pose annotations are used in the experiments. As a standard benchmark collected in a controlled indoor environment, BIWI is useful for further assessing the performance of the proposed method under constrained conditions. Together, these datasets enable a comprehensive evaluation of the proposed method under both unconstrained natural scenes and controlled indoor environments.

#### 3.1.2. Data Preprocessing

To improve the stability and generalization ability of model training, a unified data preprocessing pipeline was applied to the input images. For the 300W-LP and AFLW2000 datasets, a standard loose bounding-box cropping strategy based on facial landmarks was adopted. Specifically, the two-dimensional facial landmarks provided in the annotation files were first used to determine the minimum enclosing bounding box of the face region. The bounding box was then appropriately expanded with predefined margins to preserve sufficient facial context, and the cropped face region was subsequently resized to the required network input size. For the training set, the expansion ratio of the bounding box was randomly varied to enhance the robustness of the model to scale variations and localization perturbations, whereas a fixed bounding-box expansion setting was used during evaluation to ensure stable and reproducible testing.

After the face region was cropped, the image was uniformly resized to an input size of 224 × 224 and converted into tensor format. The input image was then normalized using the mean and standard deviation commonly adopted for ImageNet, so as to ensure consistency in the input distribution across different datasets. For the BIWI dataset, only the RGB images and their corresponding pose annotations were used in this study, and the same resizing and normalization settings were applied to maintain a consistent input format across different test datasets.

It should be noted that, since the proposed method uses cropped face images as input, perspective distortions may occur when the target face is off-center in the original image. Such distortions may slightly affect head pose estimation, especially under large head rotations or when the face is significantly displaced from the image center. Nevertheless, the loose bounding-box cropping strategy preserves sufficient facial contextual information, improves training efficiency, and ensures a consistent network input size. In addition, the randomized bounding-box expansion strategy used during training can partially alleviate the bias introduced by scale variations and non-centered faces.

The above preprocessing steps help preserve the main pose-related information in the head region while reducing the influence of background variations, scale changes, and image-quality fluctuations during both training and testing. This provides a more stable input basis for subsequent head pose feature learning. In addition, the preprocessing setting follows that of the original 6DRepNet as closely as possible, thereby ensuring a fair comparison between the improved model and the baseline.

### 3.2. Baseline 6DRepNet

This work takes 6DRepNet as the baseline network and further improves it for head pose estimation. 6DRepNet is a head pose estimation method based on a 6D rotation representation. Its core idea is to directly regress a 6-dimensional rotation representation corresponding to head pose using a convolutional neural network, and then map it to a 3D rotation matrix through an orthogonalization process. This formulation avoids the gimbal lock problem of Euler-angle representations as well as the unit-norm constraint required by quaternion-based representations. Compared with directly regressing the three Euler angles—yaw, pitch, and roll—the 6D rotation representation provides better continuity and optimization stability, and therefore shows good robustness and convergence behavior in head pose estimation tasks [24].

In the original 6DRepNet, the input face image is first fed into the backbone network to extract deep visual features, and global average pooling is then used to compress the spatial dimensions and obtain a high-level semantic representation for pose regression. Let the input image be I. The extracted feature representation is given in Equation (1):

(1) $$F=\Phi (I),\;F\in R^{C\times H\times W},$$

where Φ(⋅) denotes the backbone mapping, C represents the number of feature channels, H and W denote the spatial dimensions of the feature map, respectively. The feature map F is then subjected to global average pooling to obtain a global feature vector, as shown in Equation (2):

(2) $$z\;=\;GAP(F),\;z\;\in \;R^{C},$$

After obtaining the global feature vector, the original 6DRepNet uses a linear regression layer to map it into the 6D rotation representation space, as given in Equation (3):

(3) $$p\;=\;Wz\;+\;b,\;p\;\in \;R^{6}$$

where W and b denote the weight and bias parameters of the fully connected layer, respectively. This 6D vector does not directly correspond to Euler angles; instead, it serves as an implicit representation of the first two columns of the rotation matrix. Then, to obtain a valid rotation matrix satisfying the orthogonality constraint, the 6D rotation representation is transformed through an orthogonalization mapping, as expressed in Equation (4):

(4) $$\hat{R}=g(p),$$

where g(⋅) denotes the orthogonalization mapping from the 6D representation to the rotation matrix, and $\hat{R}\in R^{3\times 3}$ denotes the predicted rotation matrix. This mapping constructs a valid rotation matrix by normalizing and orthogonalizing the first two column vectors, thereby ensuring orthogonality of the output and maintaining good continuity during training.

During training, 6DRepNet typically adopts a geodesic loss defined on the rotation manifold to measure the angular discrepancy between the predicted rotation matrix and the ground-truth rotation matrix. Let R denote the ground-truth rotation matrix and $\hat{R}$ denote the predicted rotation matrix. The loss for a single sample is defined in Equation (5):

(5) $$d_{geo}(R,\hat{R})=\arccos\left(\frac{tr(R^{⊤}\hat{R})-1}{2}\right),$$

where $tr\left(\cdot \right)$ denotes the matrix trace operator. Compared with conventional Euclidean distance, the geodesic loss more accurately measures the true geometric discrepancy between two rotations, making it more suitable for 3D pose regression tasks.

**Figure 1:**
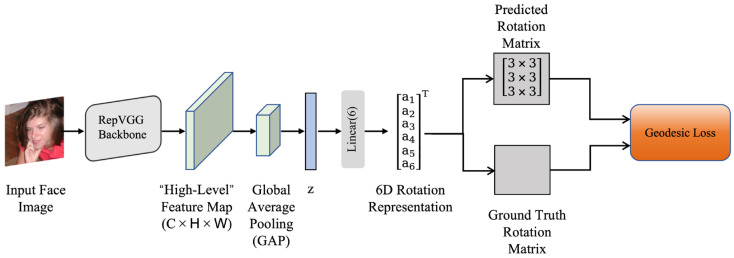
Overall architecture of the original 6DRepNet.

The overall architecture of the original 6DRepNet is illustrated in Figure 1. Given an input face image, the model first extracts deep visual features through the backbone network, then obtains a compact global semantic representation via global average pooling, and finally predicts the rotation matrix through 6D regression and orthogonalization. Owing to its simple implementation, stable training, and good representational continuity, 6DRepNet has become one of the representative baseline models for head pose estimation.

Nevertheless, the original 6DRepNet still has certain limitations. First, its regression head consists only of a single linear layer, which limits its ability to model the complex nonlinear relationship between high-level semantic features and rotation representations. Second, the network mainly relies on global features extracted by the backbone and lacks an explicit mechanism for modeling pose-sensitive regions, which may restrict prediction performance in the presence of occlusion, background interference, or large pose variations. Based on these observations, this paper further introduces a Spatial Recalibration Module, a Residual Pose Mapping Head, and a Pose-Aware Weighted Geodesic Loss on top of 6DRepNet to improve overall head pose estimation performance.

### 3.3. Improved 6DRepNet

To address the limitations of the original 6DRepNet in spatial feature modeling, pose representation mapping, and optimization for large-pose samples, this paper proposes an improved head pose estimation framework. The proposed method retains the continuous 6D rotation representation, the orthogonalization mapping to SO(3), and geodesic supervision, thereby maintaining geometric consistency during rotation regression. On this basis, lightweight improvements are introduced to the baseline model from three aspects: high-level feature enhancement, pose mapping head design, and training objective optimization.

**Figure 2:**
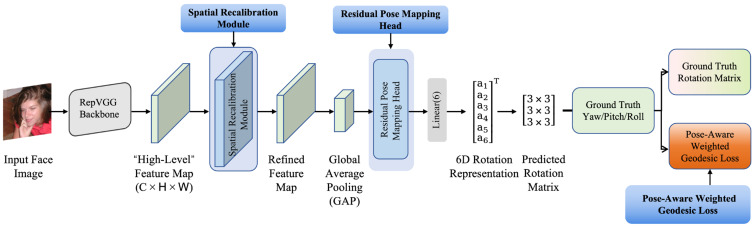
Overall architecture of the proposed geometry-aware enhanced 6DRepNet for single-RGB head pose estimation.

The overall architecture of the proposed framework is illustrated in Figure 2. Given an input face image, the model first extracts high-level semantic features using the RepVGG-B1 backbone [25]. The backbone is divided into five stages, each consisting of multiple RepVGG blocks, with the number of channels gradually increasing from 64 to 1024. The resulting feature vector is fed into subsequent modules. Each block contains convolutional layers followed by batch normalization and ReLU activation, and the backbone is initialized with ImageNet pretrained weights to accelerate convergence and improve feature representation.

After the backbone output, a Spatial Recalibration Module (SRM) is introduced to enhance the response of pose-sensitive regions and suppress background interference [26], thereby improving the spatial discriminability of high-level features. The enhanced feature map is then globally average-pooled to obtain a global feature vector, which is input to the Residual Pose Mapping Head (RPMH) for nonlinear feature mapping and refinement.

The RPMH architecture is as follows:

- The input dimension is 1024 (from the backbone output).
- The first fully connected layer maps 1024 → 512, followed by batch normalization and ReLU activation, with a dropout probability of 0.5 applied after ReLU.
- The second fully connected layer maps 512 → 256, followed by batch normalization, ReLU, and dropout.
- The output layer maps 256 → 6, corresponding to the 6D rotation representation.

This design allows RPMH to effectively capture the nonlinear relationship between high-level semantic features and rotation parameters, while maintaining training stability and enhancing generalization.

During training, a Pose-Aware Weighted Geodesic Loss is employed, which assigns higher weights to large-pose and difficult samples based on their ground-truth rotation angles, as formulated in Equation (6). The detailed definition of the loss function and the weight design are provided in Equation (6):

(6) $$L=\frac{1}{N}\sum_{i=1}^{N}w_{i}\;d_{geo}(R_{i},{\hat{R}}_{i}),$$

In summary, the proposed approach modularly enhances the original 6DRepNet along three aspects: spatial feature modeling, pose mapping capability, and training objective design [27]. These three improvements collaboratively enhance head pose estimation performance under single-RGB input and provide a foundation for subsequent module-specific experiments and validation.

### 3.4. Spatial Recalibration Module

Although 6DRepNet employs a 6D rotation representation to alleviate the discontinuity and optimization instability associated with conventional pose representations, its feature extraction process still mainly relies on the backbone to encode global semantic information, without explicitly modeling the importance of spatial locations. In head pose estimation, different spatial regions contribute unequally to pose inference. Local regions closely related to head orientation usually contain richer pose cues, whereas background textures, occluded regions, and low-response areas may weaken the discriminative power of the learned features. Therefore, an adaptive spatial recalibration mechanism is introduced at the high-level feature stage to enhance the responses of pose-sensitive regions while suppressing irrelevant interference [28].

**Figure 3:**
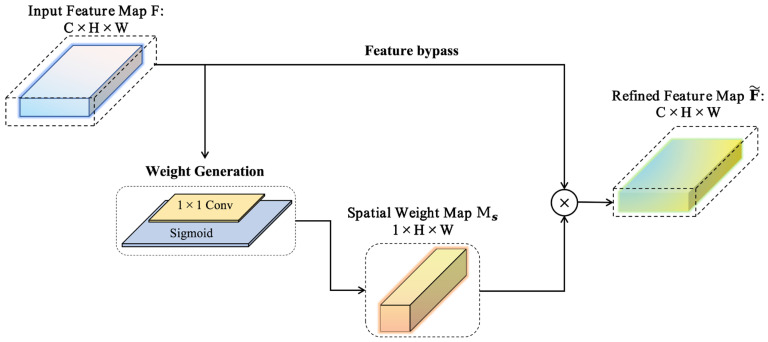
Structure of the proposed spatial recalibration module.

Motivated by this observation, a Spatial Recalibration Module is introduced after the final stage of the backbone, between the last feature map and the global average pooling layer. The module operates directly on the high-level 2D feature maps produced by the backbone under a single-RGB setting, and explicitly reweights spatial locations while preserving high-level semantic information, thereby providing more discriminative input features for subsequent pose mapping. The structure of this module is illustrated in Figure 3. This placement is chosen because the features at this stage already possess strong semantic abstraction capability while still retaining essential spatial structure, making them suitable for the explicit recalibration of pose-sensitive regions.

Let the feature map output by the backbone be given by Equation (7):

(7) $$F\in R^{C\times H\times W},$$

where C denotes the number of channels, while H and W denote the spatial height and width of the feature map, respectively. To estimate the importance of each spatial location, a 1×1 convolution is applied to the input feature map to project the original multi-channel features into a single-channel response map [29]. This process can be formulated as Equation (8):

(8) $$A={Conv}_{1\times 1}(F),$$

where $A\in R^{1\times H\times W}$ denotes the attention response map. The response values are then normalized to the range $\left[0,\;1\right]$ using the Sigmoid activation function, yielding the spatial weight map, as shown in Equation (9):

(9) $$M_{s}=\sigma (A),$$

where σ(⋅) denotes the Sigmoid function, and $M_{s}\in R^{1\times H\times W}$. After obtaining the spatial weight map, it is multiplied element-wise with the original feature map to achieve adaptive enhancement at different spatial locations. The enhanced feature representation is given in Equation (10):

(10) $$\tilde{F}=F⊙M_{s},$$

where ⊙ denotes element-wise multiplication. Since $M_{s}$ is a single-channel spatial weight map, it is broadcast across all channels, thereby imposing the same weight modulation on each channel at the same spatial location. As a result, regions with higher weights are emphasized, whereas those with lower weights are suppressed. This operation enables the network to preserve more informative local regions before the subsequent global average pooling stage. The enhanced feature map is then fed into the global average pooling layer to obtain the global feature vector $z=GAP(\tilde{F})$.

It is worth noting that the SRM is essentially a standard spatial attention mechanism, structurally similar to the spatial attention component of existing attention modules such as CBAM. For the task of single-RGB head pose estimation, we deliberately adopt spatial attention only without incorporating channel attention. On one hand, head pose information is primarily distributed across facial spatial regions, with limited dependency on channel-wise weighting. On the other hand, the spatial-only design is lightweight and computationally efficient, maintaining high-level feature discriminability while ensuring efficient processing. Experimental results indicate that this module effectively enhances the response of pose-sensitive regions and suppresses background and irrelevant areas, thereby providing reliable input features for the subsequent Residual Pose Mapping Head.

### 3.5. Residual Pose Mapping Head

In the original 6DRepNet, the high-level features extracted by the backbone are directly mapped to a 6D rotation representation through a single linear layer after global average pooling. Although this design is simple and efficient, it essentially performs only a shallow linear mapping and is therefore limited in modeling the complex nonlinear relationship between high-level semantic features and head pose parameters. This limitation becomes more pronounced under challenging conditions such as large pose variations, partial occlusions, and complex background interference.

**Figure 4:**
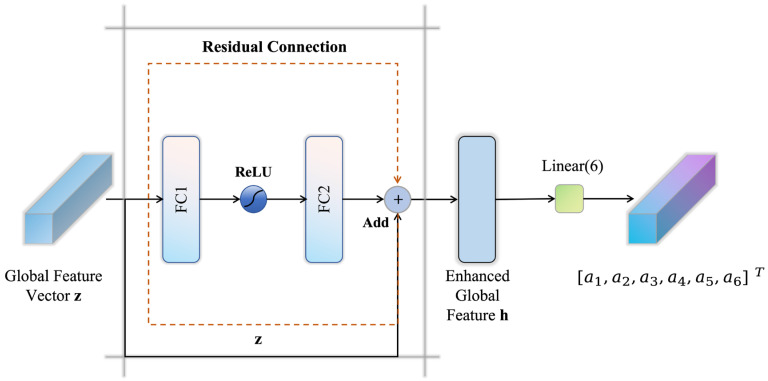
Structure of the proposed residual pose mapping head.

To address this issue, a Residual Pose Mapping Head is introduced between the global average pooling layer and the final linear regression layer to enhance the nonlinear mapping from global features to 6D pose representations [30]. Since the proposed module operates only on the low-dimensional semantic vectors obtained after global pooling, it strengthens the pose regression stage without significantly increasing model complexity. The structure of this module is illustrated in Figure 4.

Let the feature map enhanced by the Spatial Recalibration Module be $\tilde{F}\in R^{C\times H\times W}$. After global average pooling, a global feature vector $z=GAP(\tilde{F}),\;\;z\in R^{C}$ is obtained.

In the original 6DRepNet, the vector z is typically fed directly into a linear layer to generate a 6D rotation representation. In the proposed method, however, it is first processed by the Residual Pose Mapping Head for feature reconstruction and nonlinear enhancement. Specifically, this module consists of two fully connected layers, nonlinear activation functions, and a residual connection, and its computation is given in Equation (11):

(11) $$\left\{\begin{array}{c} f_{MLP}(z)=W_{2}\;\delta (W_{1}z+b_{1})+b_{2},\\ h=z+f_{MLP}(z), \end{array}\right.$$

where $W_{1}$ and $W_{2}$ denote the weight parameters of the two fully connected layers, respectively, while $b_{1}$ and $b_{2}$ represent the corresponding bias terms. δ(⋅) denotes the nonlinear activation function. In this implementation, ReLU is adopted to enhance the model’s capacity to fit complex nonlinear relationships. As indicated by the above formulation, this module does not completely replace the original global features; instead, it learns an incremental pose-related representation while preserving the original semantic information.

After being processed by the Residual Pose Mapping Head, the enhanced features are fed into the final linear regression layer, which outputs a 6D rotation representation. This process can be expressed as follows in Equation (12):

(12) $$p=W_{r}h+b_{r},$$

where $W_{r}$ and $b_{r}$ are the weight and bias parameters of the final regression layer, respectively. The predicted rotation matrix is then obtained by transforming the 6D rotation representation through an orthogonalization mapping. This process can be formulated as follows $\hat{R}=g(p)$. Where g(⋅) denotes the orthogonalization mapping from the 6D rotation representation to the rotation matrix [31].

Compared with the original single-layer linear regression head, the proposed Residual Pose Mapping Head has two main advantages. First, the two-layer perceptron together with the nonlinear activation enhances the mapping capability from high-level semantic features to the 6D pose representation, enabling the network to better capture implicit variation patterns under complex pose conditions. Second, the residual connection allows the module to preserve the original global semantic information while learning complementary pose-related representations, thereby improving training stability and mitigating the risk of feature degradation caused by deeper mappings [32].

Overall, this module does not alter the basic architecture of 6DRepNet, but instead provides a lightweight enhancement of global features at the pose regression stage. Since it mainly operates on the low-dimensional vector after global average pooling, the additional computational overhead is limited. At the same time, it effectively improves feature representation capacity and pose regression performance, thereby providing a more reliable basis for subsequent 6D rotation representation prediction [33].

### 3.6. Pose-Aware Weighted Geodesic Loss

During training, the standard geodesic loss has already been defined in Equation (5) to measure the geometric discrepancy between the predicted rotation matrix and the ground-truth rotation matrix. However, in practical training, this loss applies uniform supervision to all samples and does not distinguish the learning difficulty associated with different pose conditions. In head pose estimation, large-pose samples are usually accompanied by more pronounced appearance changes, self-occlusion, and variations in local geometric structure, making them generally more difficult to predict than near-frontal samples. If all samples are assigned equal weights in the loss function, the model tends to focus on the more abundant and easier near-frontal samples during training, which may limit its performance under complex pose conditions.

**Figure 5:**
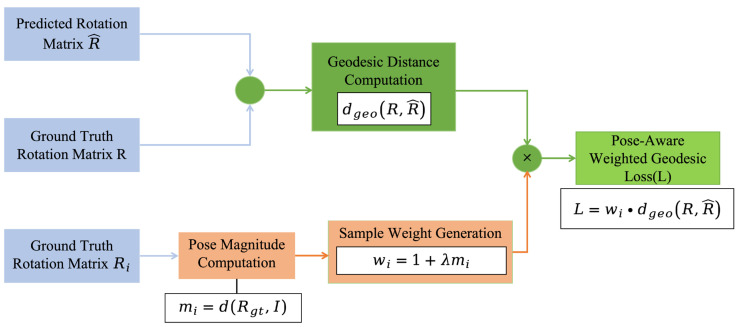
Computation process of the proposed Pose-Aware Weighted Geodesic Loss.

To alleviate this issue, a pose-aware weighting strategy is introduced on top of the standard geodesic loss, resulting in a Pose-Aware Weighted Geodesic Loss. The key idea is to assign different loss weights to individual samples according to the overall magnitude of their ground-truth pose angles, thereby increasing the optimization priority of large-pose challenging samples during training [34]. Unlike a generic sample reweighting strategy, this weighting design is derived from the rotation magnitude of the ground-truth pose and is therefore consistent with the geometric formulation of the 6D rotation representation and geodesic loss. The overall computation process of this loss is illustrated in Figure 5.

Unlike the original implementation based on Euler angles, in this work the rotation magnitude is computed directly from the ground-truth rotation matrix and the frontal zero-pose (identity matrix). This ensures consistency with the continuous 6D rotation representation and avoids the discontinuity and gimbal lock issues associated with Euler angles. Specifically, given a batch of N samples, the final Pose-Aware Weighted Geodesic Loss is defined as in Equation (13):

(13) $$L=\frac{1}{N}\sum_{i=1}^{N}w_{i}\;d_{geo}(R_{i},{\hat{R}}_{i}),$$

where $d_{geo}(R_{i},{\hat{R}}_{i})$ denotes the geodesic loss of the i sample, and $w_{i}$ represents the corresponding sample weight.

The rotation magnitude $m_{i}$ is calculated based on the geodesic distance between the ground-truth rotation matrix $R_{gt}$ and the frontal zero-pose I,$m_{i}=d\left(R_{gt},I\right)$. Where $d\left(\cdot ,\cdot \right)$ denotes the geodesic distance between two rotation matrices, as defined in Equation (14):

(14) $$d\left(R_{1},R_{2}\right)=arccos\left(\frac{trace\left(R_{1}^{⊺}R_{2}-1\right)}{2}\right),$$

Based on this, the sample weights are defined in Equation (15):

(15) $$w_{i}=1+\lambda m_{i},$$

where λ is a hyperparameter that controls the extent to which pose magnitude modulates the loss weight. In the experiments, λ is set to 0.3 so as to moderately enhance the influence of large-pose samples while maintaining training stability.

This design enables the network to pay more attention to large-pose or difficult samples during training, thereby better learning feature mappings under complex head pose conditions. The strategy does not alter the network architecture and does not introduce significant additional computational cost, but effectively compensates for sample difficulty at the optimization level. For head pose estimation, this approach reduces bias toward conventional pose samples and improves regression robustness under challenging pose conditions [35].

Combined with the previously introduced Spatial Recalibration Module (SRM) and Residual Pose Mapping Head (RPMH), the PAW Geodesic Loss strengthens constraints on large-pose and difficult samples at the optimization level: SRM enhances the response of pose-sensitive regions in high-level features, RPMH improves the nonlinear mapping from global features to 6D rotation representation, and the weighted geodesic loss adjusts sample-level weights to compensate for pose difficulty [36]. These three components jointly operate at the levels of feature modeling, pose mapping, and training optimization, collaboratively improving head pose estimation performance under single-RGB conditions.

## 4. Results

This section presents the experimental evaluation of the proposed method, including the experimental setup, evaluation metrics, overall performance comparison, ablation studies, and additional component analyses. First, the implementation details, training configuration, and evaluation protocol are introduced to ensure the reproducibility of the experiments. Then, the performance of the proposed method is compared with that of the baseline 6DRepNet on the AFLW2000 and BIWI datasets to verify its effectiveness under both unconstrained and controlled scenarios. Furthermore, the contributions of the Spatial Recalibration Module, Residual Pose Mapping Head, and Pose-Aware Weighted Geodesic Loss are systematically examined through ablation studies on both datasets. Finally, additional analyses, including performance under different pose ranges and computational complexity, are provided to further evaluate the robustness, complementarity, and efficiency of the proposed framework.

### 4.1. Experimental Setup

All experiments were implemented based on the PyTorch framework, and Adam was adopted as the optimizer [37]. The proposed method was trained on the 300W-LP dataset and evaluated on the AFLW2000 and BIWI datasets. To ensure the comparability of the experimental results, all comparative models were trained and tested under the same hardware environment and with consistent training parameter settings unless otherwise specified. Specifically, the model was trained for 80 epochs with a batch size of 16 and an initial learning rate of $1\times 10^{-4}$. For the backbone, the learning rate was set to $1\times 10^{-5}$ to reduce parameter fluctuations in the pretrained feature extraction component during training. The specific software and hardware environment, together with the main training parameter settings, are summarized in Table 1.

### 4.2. Evaluation Metrics

To comprehensively evaluate the performance of the proposed model in head pose estimation, the mean absolute errors (MAEs) of yaw, pitch, and roll are reported separately, while the overall MAE is adopted as the primary evaluation metric. Assume that the test set contains N samples. For the i sample, let $\left(yaw_{i},pitch_{i},roll_{i}\right)$ and $\left({\hat{yaw}}_{i},{\hat{pitch}}_{i},{\hat{roll}}_{i}\right)$ denote the ground-truth and predicted pose angles, respectively. The angular errors of the three pose components are defined in Equation (16):

(16) $$\left\{\begin{array}{c} E_{yaw}=\frac{1}{N}\sum_{i=1}^{N}\left|yaw_{i}-{\hat{yaw}}_{i}\right|,\\ E_{pitch}=\frac{1}{N}\sum_{i=1}^{N}\left|pitch_{i}-{\hat{pitch}}_{i}\right|,\\ E_{roll}=\frac{1}{N}\sum_{i=1}^{N}\left|roll_{i}-{\hat{roll}}_{i}\right|. \end{array}\right.$$

Based on the above definitions, the overall mean absolute error is calculated as shown in Equation (17):

(17) $$MAE=\frac{E_{yaw}+E_{pitch}+E_{roll}}{3}.$$

It should be noted that the proposed model predicts a rotation matrix $\hat{R}$ during the forward pass. During the testing stage, the predicted rotation matrix is converted into Euler angles through the corresponding decomposition procedure, and the errors in yaw, pitch, and roll are then calculated separately [38]. In this way, the reported results retain the geometric consistency of rotation-matrix-based optimization during training, while remaining directly comparable to existing head pose estimation methods.

### 4.3. Performance on AFLW2000 and BIWI

To evaluate the effectiveness of the proposed method, its performance is compared with that of the baseline 6DRepNet on the AFLW2000 and BIWI datasets. The results are reported in Table 2.

As shown in Table 2, the proposed method consistently outperforms the baseline 6DRepNet on both the AFLW2000 and BIWI datasets. On AFLW2000, the overall MAE decreases from 3.97 to 3.72, corresponding to an absolute reduction of 0.25. On BIWI, the overall MAE decreases from 3.54 to 3.26, corresponding to an absolute reduction of 0.28. These results indicate that the proposed method not only improves performance on in-the-wild data, but also provides stable gains on data collected under controlled conditions.

In terms of the individual pose components, the proposed method improves Yaw, Pitch, and Roll on both datasets. Among them, the reductions in Yaw and Roll are more pronounced, suggesting that the proposed method is particularly effective in modeling head orientation changes and overall tilt.

### 4.4. Ablation Study

To verify the effectiveness of each proposed component, ablation studies were conducted on both the AFLW2000 and BIWI datasets, with the results reported in Table 3 and Table 4. Overall, all three components provide consistent gains on both datasets, and the complete model achieves the best performance in all cases, indicating good complementarity among the proposed improvements.

After introducing the Residual Pose Mapping Head on top of the baseline model, the MAE decreases from 3.97 to 3.88 on AFLW2000 and from 3.54 to 3.41 on BIWI, indicating that enhancing the nonlinear mapping from global features to 6D pose representations is effective. When the Pose-Aware Weighted Geodesic Loss is further incorporated, the MAE decreases from 3.88 to 3.81 on AFLW2000 and from 3.41 to 3.37 on BIWI, showing that the pose-magnitude-based weighting strategy helps the model focus more on large-pose challenging samples. By contrast, adding the Spatial Recalibration Module to the Residual Pose Mapping Head yields a more pronounced improvement, reducing the MAE from 3.88 to 3.76 on AFLW2000 and from 3.41 to 3.29 on BIWI. This indicates that high-level spatial recalibration can further enhance the responses of pose-sensitive regions and improve feature discriminability. When the Residual Pose Mapping Head is disabled and only the Spatial Recalibration Module combined with the Pose-Aware Weighted Geodesic Loss is used, the MAE reaches 3.80 on AFLW2000 and 3.35 on BIWI. This indicates that even without the Residual Pose Mapping Head, the spatial recalibration and sample weighting strategies can substantially improve model performance, while the inclusion of Residual Pose Mapping Head further enhances the overall prediction accuracy.

It is worth noting that, in terms of pitch estimation, although the overall MAE shows a clear reduction, the improvement in pitch is relatively limited compared to yaw and roll. This may be attributed to the scarcity of samples with large pitch variations in the training set, as well as factors such as facial occlusions or illumination changes. Future work could consider data augmentation for extreme pitch samples or assigning higher weights to large-pitch samples in the pose-aware weighted geodesic loss to further enhance model performance along the pitch axis.

When all three improvements are introduced simultaneously, the model achieves the best results on both datasets, with MAEs of 3.72 on AFLW2000 and 3.26 on BIWI. These results indicate that the Spatial Recalibration Module, Residual Pose Mapping Head, and Pose-Aware Weighted Geodesic Loss provide complementary benefits from three aspects—spatial feature modeling, pose representation mapping, and training objective optimization—thereby jointly improving head pose estimation performance.

### 4.5. Comparison with Standard Attention Modules

To further examine whether the performance improvement comes from the proposed spatial recalibration design rather than from the simple introduction of a generic attention mechanism, we compare the proposed Spatial Recalibration Module with two representative attention modules: the Squeeze-and-Excitation (SE) block and the Convolutional Block Attention Module (CBAM). The SE block mainly performs channel-wise feature recalibration, whereas CBAM sequentially applies channel attention and spatial attention to refine feature representations.

For a fair comparison, all attention modules are inserted at the same position, namely after the final stage of the RepVGG backbone and before the global average pooling layer. Except for the attention module, all other components are kept unchanged, including the RepVGG backbone, the Residual Pose Mapping Head, the Pose-Aware Weighted Geodesic Loss, the training and testing datasets, and the training parameter settings. As described above, the SRM is designed to enhance pose-sensitive spatial regions in high-level feature maps, the RPMH improves the nonlinear mapping from global features to the 6D rotation representation, and the PAWGL strengthens the optimization of challenging large-pose samples.

As shown in Table 5, introducing the SE module alone does not lead to consistent performance improvement. On the AFLW2000 dataset, the MAE of the SE module is 3.82, which is slightly higher than that of the variant without an attention module, whose MAE is 3.81. On the BIWI dataset, the SE module obtains an MAE of 3.37, which is almost the same as that of the variant without attention. This indicates that channel-wise feature recalibration alone has limited ability to model pose-sensitive spatial regions for head pose estimation.

In contrast, CBAM achieves better results than SE, as it incorporates both channel attention and spatial attention. Specifically, CBAM reduces the MAE to 3.78 on AFLW2000 and 3.33 on BIWI. These results suggest that spatial attention plays a useful role in head pose estimation by helping the model enhance pose-related regions.

Compared with SE and CBAM, the proposed SRM achieves the lowest overall errors on both datasets. The MAE is reduced to 3.72 on AFLW2000 and 3.26 on BIWI. Compared with the variant without an attention module, SRM reduces the MAE by 0.09 and 0.11 on the two datasets, respectively. These results demonstrate that the proposed SRM does not simply benefit from the use of a generic attention mechanism. Instead, by explicitly enhancing pose-sensitive spatial responses in high-level feature maps before global average pooling, SRM more effectively improves the performance of single-RGB head pose estimation.

Overall, SE mainly focuses on channel-wise feature selection, while CBAM adopts a more general channel-spatial attention mechanism. In comparison, the proposed SRM employs a more direct spatial recalibration strategy, which enhances pose-related spatial responses while keeping the module lightweight. Therefore, this comparison further verifies the effectiveness and task-specific suitability of the proposed SRM for head pose estimation.

### 4.6. Component Evaluation

To further evaluate the proposed method, additional analyses are conducted from two perspectives: performance under different pose ranges and computational complexity.

#### 4.6.1. Performance Under Different Pose Ranges

**Figure 6:**
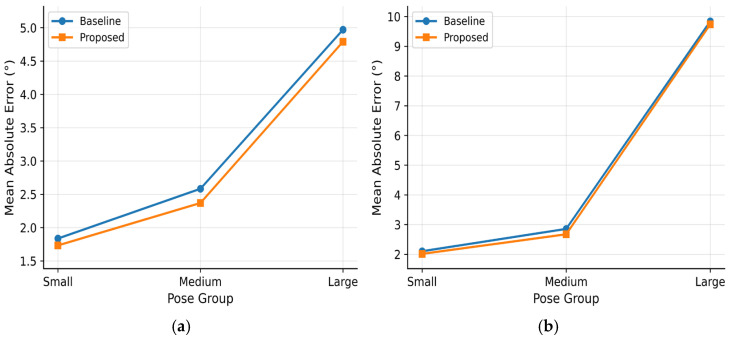
Mean absolute error comparison across different pose groups: (**a**) AFLW2000 dataset; (**b**) BIWI dataset.

To further evaluate the adaptability of the proposed method under different pose ranges, the test samples in the AFLW2000 and BIWI datasets are divided into different pose groups according to pose magnitude, and the MAE results of the baseline model and the proposed method are compared within each group. Figure 6 illustrate the corresponding error comparisons on the AFLW2000 and BIWI datasets, respectively.

As shown in Figure 6, the proposed method consistently outperforms the baseline model across all three pose groups on both datasets, with a more pronounced reduction in error in the large-pose group. This indicates that the proposed method not only improves estimation accuracy under conventional pose conditions, but also maintains good robustness when pose magnitude becomes larger and appearance variations become more complex. This observation is consistent with the design motivation of the Pose-Aware Weighted Geodesic Loss, suggesting that the pose-magnitude-based weighting strategy can effectively enhance the model’s focus on large-pose challenging samples during training.

Overall, Figure 6a,b exhibit similar performance trends, further demonstrating that the proposed method maintains stable advantages under different data distributions and pose ranges. These results indicate that the joint design of the Spatial Recalibration Module, Residual Pose Mapping Head, and Pose-Aware Weighted Geodesic Loss effectively improves the model’s adaptability to complex pose samples, thereby enhancing overall head pose estimation performance.

#### 4.6.2. Computational Complexity Analysis

To evaluate the balance between performance improvement and computational cost of the proposed method, we compared the baseline model and the full model in terms of the number of parameters (Params), floating-point operations (FLOPs), and inference speed (FPS), as shown in Table 6. All measurements were conducted under the same hardware and software environment described in Table 1, using 224 × 224 RGB face crops as network inputs. The FPS was calculated to evaluate the forward inference efficiency of the model.

Compared with the baseline 6DRepNet, the proposed method increases the number of parameters from 31.64 M to 31.91 M, corresponding to only a 0.85% increase. The FLOPs increase slightly from 4.32 G to 4.33 G, with an increase of only 0.23%. Although the FPS decreases from 84.68 to 78.54, the proposed method still maintains real-time inference capability. These results demonstrate that the introduced modules bring only marginal additional computational overhead while providing consistent accuracy improvements, indicating a favorable balance between estimation performance and computational complexity.

### 4.7. Comparative Experiments on Different Head Pose Estimation Models

To further evaluate the relative performance of the proposed method in head pose estimation, it is compared with several representative methods. It should be noted that, since differences may exist across studies in terms of training data, preprocessing strategies, and experimental protocols, the results in this section are mainly intended for horizontal reference, so as to provide a comprehensive evaluation of the competitiveness of the proposed method on the AFLW2000 and BIWI datasets.

As shown in Table 7, the proposed method demonstrates strong competitiveness on both the AFLW2000 and BIWI datasets. On AFLW2000, the proposed method achieves errors of 3.36°, 4.69°, and 3.11° for Yaw, Pitch, and Roll, respectively, with an overall MAE of 3.72°. Compared with the reproduced baseline 6DRepNet, the overall MAE is reduced from 3.97° to 3.72°. On BIWI, the proposed method achieves errors of 3.16°, 4.19°, and 2.44° for Yaw, Pitch, and Roll, respectively, with an overall MAE of 3.26°. Compared with the reproduced baseline 6DRepNet, the overall MAE is reduced from 3.54° to 3.26°.

Further comparison with representative methods, including FAN, HopeNet, FSA-Net, LSR, MFDNet, DirectMHP, and Img2Pose, shows that the proposed method achieves competitive overall errors on both datasets. These results indicate that the proposed method not only performs well under natural-scene conditions and complex pose distributions, but also maintains stable performance on data collected under controlled environments.

Taken together, the results on the two datasets demonstrate that the proposed method generalizes well across different data distributions and imaging conditions. Combined with the dual-dataset ablation studies presented earlier, this further indicates that the performance gain does not arise from a single component, but from the joint contribution of the Spatial Recalibration Module, Residual Pose Mapping Head, and Pose-Aware Weighted Geodesic Loss from the perspectives of spatial feature modeling, pose representation mapping, and training objective optimization.

### 4.8. Visual Analysis

**Figure 7:**
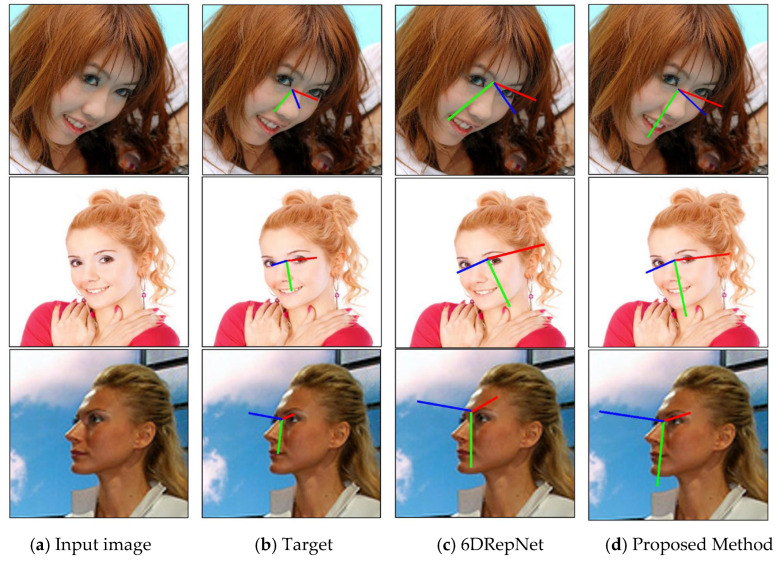
Qualitative comparison of head pose estimation on the AFLW2000 dataset. Each row contains four columns: (**a**) input image, (**b**) ground-truth pose, (**c**) 6DRepNet prediction, and (**d**) proposed method prediction. Pose axes are superimposed on the face to visualize head orientation. The red, green, and blue lines denote the x-, y-, and z-axes of the head pose coordinate system, respectively, which are used to visualize yaw, pitch, and roll variations. The proposed method produces predictions closer to the ground truth, particularly under large pose variations.

**Figure 8:**
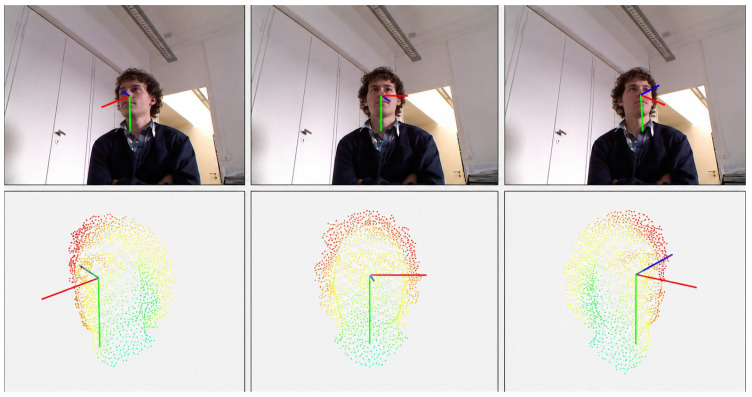
SRM attention heatmaps of the proposed method on the BIWI dataset. The heatmaps indicate the facial regions the model attends to, providing interpretability for the Spatial Recalibration Module. Warmer colors indicate higher attention responses, whereas cooler colors indicate lower attention responses. The red, green, and blue lines denote the x-, y-, and z-axes of the head pose coordinate system, respectively, which are used to visualize yaw, pitch, and roll variations. The attention is focused on key facial features such as the eyes, nose, and contours, while background regions show lower responses.

To provide a comprehensive qualitative evaluation of the proposed method under various head pose conditions, representative test samples from the AFLW2000 and BIWI datasets were selected for visualization, as shown in Figure 7 and Figure 8.

In Figure 7, each row presents a four-column comparison: (a) input image, (b) ground-truth pose, (c) prediction of the original 6DRepNet, and (d) prediction of the proposed method. Pose axes are superimposed on the facial region to facilitate direct inspection of yaw, pitch, and roll variations. Compared with the baseline 6DRepNet, the proposed method produces predictions that are closer to the ground truth, particularly for samples with large yaw, pitch, or roll angles, demonstrating more stable and accurate estimation under challenging poses. These observations highlight the effectiveness of the Spatial Recalibration Module and the Residual Pose Mapping Head in enhancing pose-sensitive feature representations and improving the nonlinear mapping from global features to 6D rotation outputs.

As shown in Figure 8, it presents SRM attention heatmaps for representative BIWI samples. The attention maps clearly indicate the facial regions the model focuses on during head pose estimation, emphasizing key areas such as the eyes, nose, and facial contours while suppressing responses from irrelevant background regions. This visualization provides interpretability for the Spatial Recalibration Module, demonstrating that the model effectively attends to pose-sensitive regions and produces reliable predictions under controlled conditions. For near-frontal samples, the pose axes remain stable, whereas for laterally rotated or downward head poses, the axes adjust accordingly, reflecting the model’s adaptability to varying head orientations.

Overall, these qualitative results show that the proposed method not only achieves improved head pose estimation compared with the baseline but also provides interpretable predictions by focusing on key facial regions. The combined effect of the Spatial Recalibration Module, Residual Pose Mapping Head, and Pose-Aware Weighted Geodesic Loss enables the model to generate consistent and accurate head pose estimates across diverse pose conditions, demonstrating both the effectiveness and interpretability of the proposed approach.

## 5. Conclusions

This paper proposes an improved method for single-RGB head pose estimation to address the limitations of 6DRepNet in high-level spatially sensitive region modeling, global feature-to-6D rotation representation mapping, and the optimization of large-pose challenging samples. While preserving the advantages of the 6D continuous rotation representation and geometry-consistent optimization, the proposed method introduces a Spatial Recalibration Module, a Residual Pose Mapping Head, and a Pose-Aware Weighted Geodesic Loss to enhance the baseline model from three aspects: high-level spatial feature modeling, pose representation mapping, and training objective optimization. Experimental results show that the proposed method consistently outperforms the baseline 6DRepNet on both the AFLW2000 and BIWI datasets, reducing the overall MAE from 3.97 to 3.72 and from 3.54 to 3.26, respectively. Ablation studies, pose-range analysis, and complexity analysis further verify the effectiveness and complementarity of the proposed components, showing that the method achieves stable performance gains while maintaining relatively high real-time efficiency.

Despite the promising results, several limitations remain. First, the improvement in Pitch is relatively limited, indicating that the current model still has room for improvement in modeling certain vertical pose variation patterns. In addition, the datasets used in this study include 300W-LP for training, and AFLW2000 and BIWI for testing, covering both in-the-wild and controlled scenarios for head pose estimation. To further evaluate the model’s generalization ability on unseen data distributions, cross-dataset experiments are necessary, i.e., evaluating the model on datasets not used during training to assess robustness across different data distributions. Due to current experimental resource limitations, such cross-dataset evaluations have not yet been conducted. Nevertheless, it is expected that the proposed method, with the combined effects of the Spatial Recalibration Module, Residual Pose Mapping Head, and Pose-Aware Weighted Geodesic Loss, would maintain stable performance and accurate head pose estimation. Future work will include systematic testing on additional unseen datasets to fully assess the model’s generalization capability. Future work may further improve robustness and generalization by incorporating richer data augmentation strategies, stronger regularization methods, or larger-scale training datasets [40].

## Figures and Tables

**Table 1:** Training environment and parameters.

Environment/Parameter	Setting
Operating System	Ubuntu 22.04.3 LTS
GPU	NVIDIA RTX 3090, NVIDIA Corporation, Santa Clara, CA, USA
CPU	Intel Xeon(R) Gold 6330, Intel Corporation, Santa Clara, CA, USA
CUDA	12.1, NVIDIA Corporation, Santa Clara, CA, USA
PyTorch	2.1.2
Python	3.10.8
Optimizer	Adam
Initial learning rate	$1\;\times \;10^{-4}$
Backbone learning rate	$1\;\times \;10^{-5}$
Epochs	80
Batch size	16
Training dataset	300W-LP
Test datasets	AFLW2000, BIWI

**Table 2:** Overall results on the AFLW2000 and BIWI datasets.

Method	AFLW2000				BIWI			
	Yaw	Pitch	Roll	MAE	Yaw	Pitch	Roll	MAE
Baseline (6DRepNet)	3.63	4.91	3.37	3.97	3.46	4.48	2.68	3.54
Proposed Method	3.36	4.69	3.11	3.72	3.16	4.19	2.44	3.26

**Note:** Lower values of Yaw, Pitch, Roll, and MAE indicate better performance.

**Table 3:** Results of ablation experiments on the AFLW2000 dataset. RPMH denotes Residual Pose Mapping Head, PAWGL denotes Pose-Aware Weighted Geodesic Loss, and SRM denotes Spatial Recalibration Module.

No.	RPMH	PAWGL	SRM	Yaw (°)	Pitch (°)	Roll (°)	MAE
1				3.63	4.91	3.37	3.97
2	√			3.50	4.85	3.31	3.88
3	√	√		3.46	4.71	3.26	3.81
4		√	√	3.42	4.76	3.22	3.80
5	√		√	3.38	4.72	3.19	3.76
6	√	√	√	3.36	4.69	3.11	3.72

**Note:** Lower values of Yaw, Pitch, Roll, and MAE indicate better performance.

**Table 4:** Results of ablation experiments on the BIWI dataset. RPMH denotes Residual Pose Mapping Head, PAWGL denotes Pose-Aware Weighted Geodesic Loss, and SRM denotes Spatial Recalibration Module.

No.	RPMH	PAWGL	SRM	Yaw (°)	Pitch (°)	Roll (°)	MAE
1				3.46	4.48	2.68	3.54
2	√			3.31	4.39	2.55	3.41
3	√	√		3.28	4.33	2.51	3.37
4		√	√	3.24	4.28	2.53	3.35
5	√		√	3.19	4.22	2.47	3.29
6	√	√	√	3.16	4.19	2.44	3.26

**Note:** Lower values of Yaw, Pitch, Roll, and MAE indicate better performance.

**Table 5:** Comparison with standard attention modules on the AFLW2000 and BIWI datasets.

Attention Module	AFLW2000				BIWI			
	Yaw	Pitch	Roll	MAE	Yaw	Pitch	Roll	MAE
None	3.46	4.71	3.26	3.81	3.28	4.33	2.51	3.37
SE	3.48	4.73	3.25	3.82	3.27	4.34	2.50	3.37
CBAM	3.41	4.73	3.20	3.78	3.23	4.28	2.48	3.33
SRM	3.36	4.69	3.11	3.72	3.16	4.19	2.44	3.26

**Note:** Lower values of Yaw, Pitch, Roll, and MAE indicate better performance. All variants use the same RepVGG backbone, Residual Pose Mapping Head, and Pose-Aware Weighted Geodesic Loss. Only the attention module is changed.

**Table 6:** Complexity comparison between the baseline model and the proposed method.

Model	Params (M)	FLOPs (G)	FPS
6DRepNet	31.64	4.32	84.68
Proposed Method	31.91	4.33	78.54

**Note:** Lower values are better for Params and FLOPs, whereas higher values are better for FPS.

**Table 7:** Performance comparison of different methods on the AFLW2000 and BIWI datasets.

Method	AFLW2000				BIWI			
	Yaw	Pitch	Roll	MAE	Yaw	Pitch	Roll	MAE
FAN [39]	6.36	12.30	8.17	9.12	8.53	7.48	7.63	7.89
HopeNet (α = 2)	6.47	6.56	5.44	6.18	5.17	6.98	3.39	5.18
WHENET	6.47	6.56	5.44	6.16	4.81	6.60	3.27	4.89
FSA-Net	4.50	6.08	4.64	5.07	4.27	4.96	2.76	4.00
WHENet	4.44	5.75	4.31	4.83	3.60	4.10	2.73	3.48
LSR	4.26	5.27	3.89	4.47	4.29	3.09	3.18	3.52
MFDNet [7]	4.30	5.16	3.69	4.38	3.40	4.68	2.77	3.62
DirectMHP	2.99	5.35	3.77	4.04	3.57	5.47	4.02	4.35
6DRepNet	3.63	4.91	3.37	3.97	3.46	4.48	2.68	3.54
Img2Pose	3.43	5.03	3.28	3.91	4.57	3.55	3.24	3.79
Proposed Method	3.36	4.69	3.11	3.72	3.16	4.19	2.44	3.26

**Note:** Lower values of Yaw, Pitch, Roll, and MAE indicate better performance.

## Data Availability

The datasets used in this study, including 300W-LP, AFLW2000, and BIWI, are publicly available from the sources cited in the manuscript.
